# Differential Expression of Complement Pathway Components in Unexplained Infertility Versus Male Factor Infertility: Insights from an Exploratory Pilot Study

**DOI:** 10.3390/ijms262010168

**Published:** 2025-10-19

**Authors:** Edwina Brennan, Marya K. E. A. Radhi, Zainab A. A. H. Husain, Thozhukat Sathyapalan, Abu Saleh Md Moin, Alexandra E. Butler, Stephen L. Atkin

**Affiliations:** 1 School of Medicine, Royal College of Surgeons in Ireland Bahrain, Adliya 15503, Bahrain; 21200448@rcsi.com (M.K.E.A.R.); 21200249@rcsi.com (Z.A.A.H.H.); amoin@rcsi.com (A.S.M.M.); abutler@rcsi.com (A.E.B.); satkin@rcsi.com (S.L.A.); 2 Academic Endocrinology, Diabetes and Metabolism, Hull York Medical School, University of Hull, Hull HU6 7RU, UK; Thozhukat.Sathyapalan@hyms.ac.uk

**Keywords:** complement pathways, infertility, unexplained infertility, properdin, C4, C8, C3adesArg

## Abstract

Complement (C) proteins have been linked to infertility and reproductive outcomes. This study was undertaken to determine the association of complement proteins in non-obese women before in vitro fertilization (IVF) with unexplained infertility (UI) compared to women with male factor infertility (MFI) as controls. We hypothesized that complement protein factors may provide evidence for the underlying mechanism in UI. In this exploratory pilot study, 25 women (UI = 14 and MFI = 11) undergoing IVF had blood drawn on day 21 of the luteal phase. Slow Off-rate Modified Aptamer (SOMA)-scan plasma protein measurement was undertaken for 25 complement pathway-related proteins. Student’s *t*-test was used to compare group means and Pearson’s correlations to examine relationships with complement proteins. Baseline demographics and hormonal parameters did not differ between groups, and parameters of the response following IVF did not differ. In the UI group compared to the MFI group, there were lower levels of properdin (*p* = 0.03) that may reduce endometrial receptivity and impact follicular development, lower C3a anaphylatoxin des arginine (C3adesArg) (*p* = 0.02) that may reduce endometrial vascularity, lower C4 (C4) (*p* = 0.04), indicating reduced alternate pathway activation, and lower C8 (C8) (*p* = 0.04) that also may affect the endometrium. In UI alone, properdin negatively correlated with high-density lipoprotein cholesterol (HDL-c), and C8 positively correlated with thyroid-stimulating hormone (TSH) and Free-triiodothyronine (Free-T3) (*p* < 0.05). These preliminary findings indicate reduced complement activity among UI women, warranting further mechanistic investigation.

## 1. Introduction

Infertility is the inability to conceive after twelve months of regular, unprotected sexual intercourse and affects 10–15% of couples [1]. There are several reasons for infertility, which include both male and female factors. Male factors alone, defined as the presence of at least one abnormality in semen analysis or inability to adequately deliver semen due to sexual or ejaculatory issues [2], are responsible for 20–30% of the infertility cases, whereas female factors are responsible for 37% of the cases. Female factors of infertility may include ovulatory disorders, endometriosis, adhesions, fallopian tube abnormalities, hormonal disturbances and uterine or cervical abnormalities [3,4]. A challenging type of infertility, which affects 30% of couples, is unexplained infertility (UI), defined as the inability to conceive in couples younger than 40 years. UI couples are diagnosed when they have infertility, normal ovarian function, tubal patency, and no pelvic, uterine, or cervical abnormalities, in addition to normal testicular function and ejaculation. All investigations must be negative before reaching a diagnosis of unexplained infertility; therefore, it is a diagnosis of exclusion [5].

**Figure 1:**
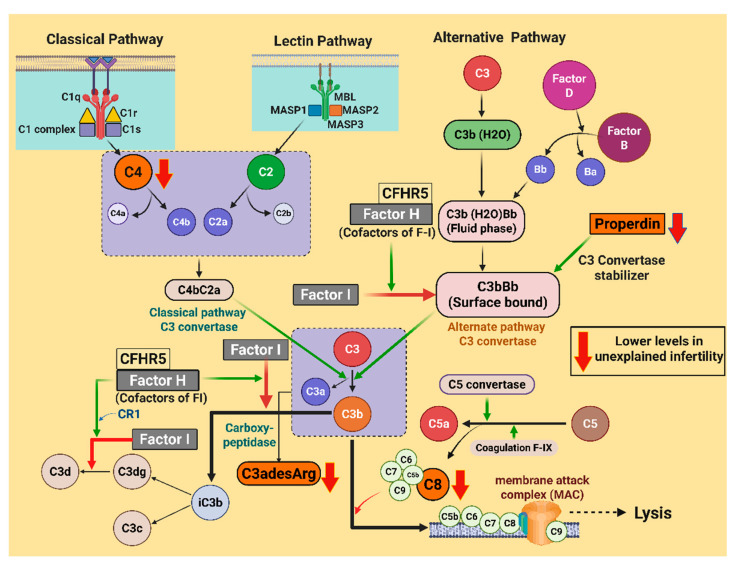
A schematic illustrating the initiating proteins of the classical, alternative, and lectin complement cascades. The green arrows in the illustration indicate the enzymatic activity or positive regulation, whereas the red arrows indicate the inhibition of pathways. Downward red arrows indicate the lower levels of the proteins (C4, properdin, C3adesArg, and C8) in the unexplained infertility (UI) cases. C1–C9, complement component 1–9; MBL, mannose-binding lectin; MASP, mannose-associated serine protease; C4a, C4 anaphylatoxin; C4bC2a and C3bBb, active C3 convertase; CFHR5, complement factor H-related protein 5; CR1, complement receptor 1; C3a, C3 anaphylatoxin; C5a, C5 anaphylatoxin; C3adesArg, C3a anaphylatoxin des arginine; and iC3b, inactivated C3b. The illustration was created using BioRender.com (with publication license).

The complement system is a critical part of the innate immune system. Combating infection and maintaining homeostasis, the completement system consists of a cascade of over thirty collaborative complement (C) proteins that, when activated, enhance phagocytosis and coordinate inflammatory mediators [6]. Studies in animal [7] and human ovaries [8] suggest that the complement system may play a role in homeostasis and tissue remodeling and therefore may be implicated in ovarian dysfunction and infertility. Furthermore, the presence of complement proteins in human follicular fluid suggests their involvement in oocyte maturation [9]. The complement system is activated through three different pathways: classical, lectin, and alternative pathways. The classical pathway is activated by C1 and the lectin pathway by mannose-binding lectin (MBL). The classical and lectin pathways converge where the cleavage of C2 and C4 generates C4bC2a, active C3 convertase. The alternative pathway gives rise to a distinct C3 convertase, C3bBb, formed through the spontaneous hydrolysis of C3 that is Factor B and Factor D-dependent. The resulting active C3 convertase of each pathway is cleaved to C5, which is subsequently cleaved yielding terminal proteins C5b-C9 that polymerize to form the potent Membrane attack complex (MAC) [10]. A key positive regulator of the complement system is properdin, which stabilizes alternative pathway C3 convertase. Conversely, Factor I and its cofactor, Factor H, are negative regulators inhibiting alternative pathway initiation and terminal complement cascade activation [11] (Figure 1).

Research studies suggest that complement proteins may contribute to endometrial receptivity, such as C3 [12]; implantation and placentation, such as C1q [13,14]; maternal–fetal immune tolerance, such as C5b-C9 [15]; and angiogenesis and tissue remodeling [16]. In women with subclinical and premature ovarian failure and UI, high concentrations of complement breakdown products have been reported [17]. In a study of obese patients with UI, higher Factor D and lower C5 anaphylatoxin (C5a) levels in the serum were markers of a successful in vitro fertilization (IVF) outcome [18]. Furthermore, women diagnosed with endometriosis showed significantly higher levels of C3 in peritoneal fluid and serum compared with women with no endometriosis [19]. The success of implantation also relies on several essential modifications in the adaptive immune system to create an environment that supports fetal development while preventing immune rejection of the embryo [20]. Complement proteins may also differ in the endometrial tissue of women during different stages of the menstrual cycle, including the proliferative, early to mid-secretory phase, and the late secretory phase [21]. Elevated levels of C3 production are reported in the luteal phase, particularly in glandular epithelial cells, versus the proliferative phase, and women undergoing hormonal progesterone therapy had increased endometrial cell concentrations of C3 compared to women who had not previously taken any hormonal therapies [21], suggesting that progesterone may modulate C3 in the luteal phase.

Increasingly recognized for its involvement in reproductive processes, including ovulation, fertilization, implantation and immune regulation during early pregnancy, disruptions or imbalances in the complement system may contribute to reproductive failure. While some research on the role of complement activation in reproductive disorders exists, to our knowledge, only two studies examine complement proteins, specifically C3 and C4, in those with UI [9,22], while no case–control study has been published to date. Gaining a deeper understanding of the role of the complement system in reproductive dysregulation could help to identify novel diagnostic markers, ultimately enabling more precise and individualized treatment strategies for patients struggling with UI. This study aimed to compare the expression of complement protein factors to determine whether they differed in women undergoing IVF with either UI or male factor infertility (MFI) as a control population, specifically on day 21 of the luteal phase, to circumvent reported differences in complement protein levels during the menstrual cycle. We hypothesized that complement protein factors may provide evidence for the underlying mechanism in UI.

## 2. Results

### 2.1. Patient Characteristics

There were no significant differences found between the MFI and UI groups in terms of age, body mass index (BMI), Homeostatic Model Assessment of Insulin Resistance (HOMA-IR), and serum lipids. C-reactive protein (CRP) and white cell count (WCC), as indicators of inflammation and infection, were within the normal range and similar for both MFI and UI. There were no significant differences found between the MFI and UI groups with regard to levels of reproductive hormones, number of positive pregnancy tests, eggs retrieved, and embryos created, including the G3D3, which are the top-quality embryos at day 3 as per the Alpha consensus (23). In addition, the fertility rates and the live birth rates did not differ between the two groups (Table 1).

### 2.2. Complement Proteins

**Figure 2:**
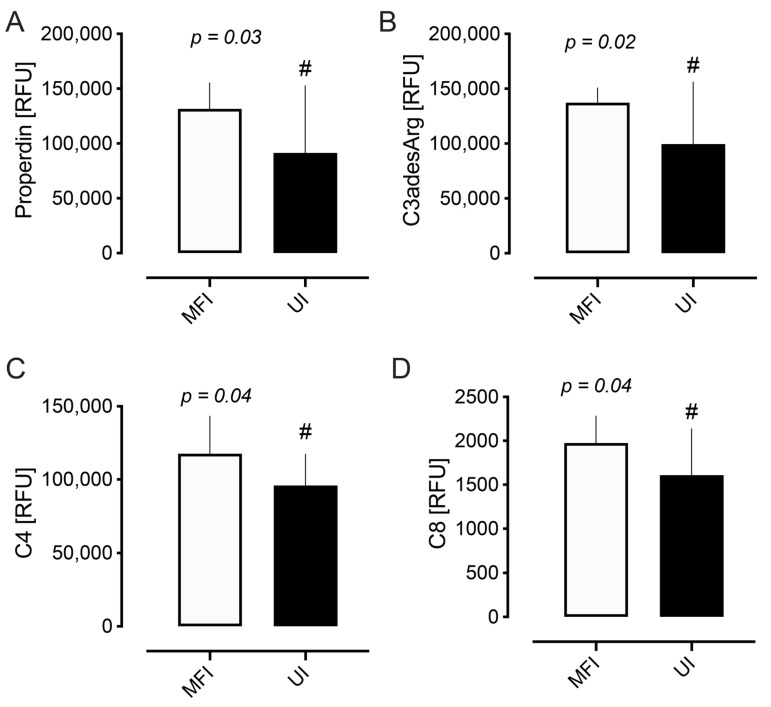
Complement pathway proteins that differed between male factor infertility (MFI) and unexplained infertility (UI). Properdin (*p* = 0.03) (**A**), C3a anaphylatoxin des arginine (C3adesArg) (*p* = 0.02) (**B**), C4 (*p* = 0.04) (**C**), and C8 (*p* = 0.04) (**D**) were lower in the UI cohort. # *p* < 0.05. Data is presented as mean (SD). Protein levels are reported as Relative Fluorescence Units (RFU).

Analysis of the complement plasma proteins showed several significant differences between the UI and MFI cohorts. Mean levels of properdin (91,387 RFU vs. 131,606 RFU, *p* = 0.03), C3a anaphylatoxin des arginine (C3adesArg) (99,554 RFU vs. 137,187 RFU, *p* = 0.02), C4 (95,901 RFU vs. 117,538 RFU, *p* = 0.04), and C8 (1609 RFU vs. 1974 RFU, *p* = 0.04) were lower in the UI versus the MFI cohort, as shown in Table 2 and Figure 2. The remaining complement proteins showed no differences between the two cohorts (Table 2).

### 2.3. Correlation Analysis

**Figure 3:**
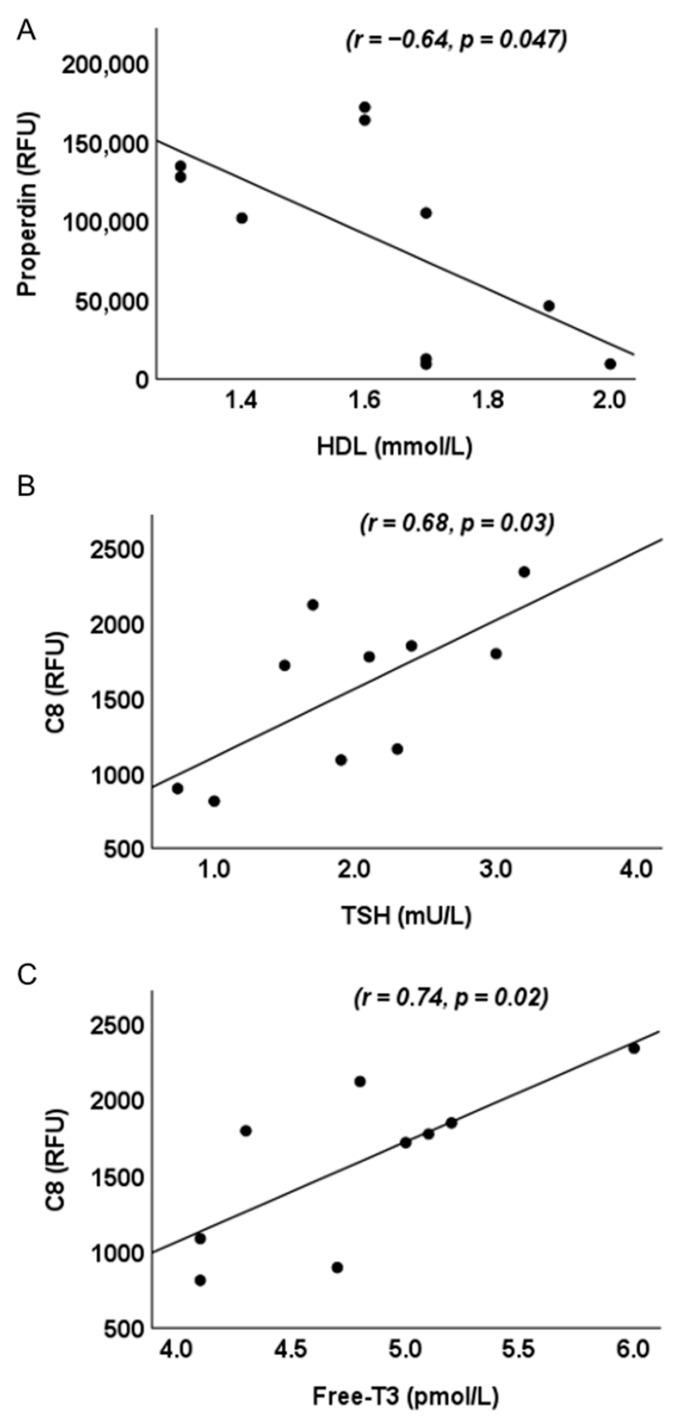
Significant correlations between complement pathway proteins and biochemical and hormonal parameters in unexplained infertility (UI). Properdin and high-density lipoprotein cholesterol (HDL-c) (r = −0.64, *p* = 0.047) (**A**), C8 and thyroid-stimulating hormone (TSH) (r = 0.68, *p* = 0.03) (**B**), and C8 and Free-triiodothyronine (Free-T3) (r = 0.74, *p* = 0.02) (**C**). Protein levels are reported as Relative Fluorescence Units (RFU).

We examined relationships between complement proteins, those that differed significantly between MFI and UI, and biochemical and hormonal parameters. In UI alone, properdin negatively correlated with high-density lipoprotein (HDL-c) (r = −0.64, *p* = 0.047) and C8 positively correlated with thyroid-stimulating hormone (TSH) (r = 0.68, *p* = 0.03) and Free-triiodothyronine (Free-T3) (r = 0.74, *p* = 0.02) (Figure 3). No significant correlations were observed between C3adesArg and C4 and measured biochemical or hormonal parameters in UI. Within the MFI cohort, no significant correlations were observed between complement proteins and any biochemical or hormonal parameter.

## 3. Discussion

The complement system, responsible for combating infections and maintaining homeostasis, is a critical part of the innate immune system that has been suggested to be implicated in ovarian dysfunction and infertility. However, to date, there is limited research in the literature on the role of complement factors in infertility. In this comprehensive study of the complement pathway proteins in UI compared to MFI, we found that properdin, C3adesArg, C4, and C8 were lower in the UI group. In the UI cohort alone, properdin was found to negatively correlate with HDL-c levels while C8 positively correlated with TSH and Free-T3 levels. To our knowledge, this is the first case–control study examining complement proteins in those with UI, and although preliminary, these novel findings suggest the possibility of hypofunction of complement activation in UI that may contribute to its pathogenesis.

Properdin is an important positive regulator of the alternative complement system. The larger cleavage product of C3, C3b, complexes with Factor B, which is subsequently activated by Factor D into active C3 convertase [23]. Through binding of C3b, as well as Factor B and Bb [24], properdin stabilizes C3 convertase by increasing its half-life 5–10 fold [25]. Properdin is secreted by monocytes, macrophages, and T lymphocytes and, in the endometrium, increases the proliferation of endometrial cells and has been implicated in endometriosis [26] via its upregulation of C3 [27]. In epithelial cells, properdin has been shown to have an early anti-inflammatory and late pro-inflammatory effect [28]. Through stabilizing active convertases on cell surfaces, properdin promotes platelet–granulocyte aggregation contributing to thromboinflammation [29]. Properdin levels were previously shown to be directly proportional to Anti-Müllerian Hormone (AMH) levels [30], raising the suggestion that there is a relationship with ovarian function and follicular development. Although there are no studies in humans, results from animal studies provide support for the claim. Equine follicular fluid properdin levels absent in the pre-deviation stage of follicular development were significantly upregulated during deviation and impending ovulation stages, suggesting a role in not only the selection of the dominant follicle but also in ovulation [31]. Similarly, in a porcine model, properdin was associated with follicular growth [32]. Our data indicate lower properdin levels in UI, suggesting that there may be a decrease in the alternative pathway; however, since the levels of C3b were not different in UI, this may conversely indicate that there is no functional impact. We did not observe any correlations between AMH and properdin levels. Instead, we found that properdin negatively correlated with HDL-c levels in those with UI where no such correlation was observed in those with MFI. HDL-c is considered to have antioxidative properties and in those with UI, oxidative stress has been suggested to be a causative factor [33]. Though some studies have found a negative association between HDL-c and infertility, [34] others have found none [35], while animal studies have shown that reduced HDL-c levels restored fertility [36]. It is unclear what impact a relative decrease in properdin would have as, to date, deficiency in humans is associated only with an increased susceptibility to meningitis infection [29]. However, its relative decrease in UI may be a marker for a dysregulated endometrial microenvironment that may be detrimental to embryo implantation. Additionally, it may be a marker of defective folliculogenesis and ovulation, as proposed by animal studies [31,32], leading to subtle effects in those with UI.

C3, the central component of the complement system, is cleaved to C3 anaphylatoxin (C3a) and C3b. C3a binds to its G protein-coupled C3a receptor, inducing a localized inflammatory response [37], and is subsequently converted by carboxypeptidase action to C3adesaArg. C3adesArg, also called acylation-stimulating protein (ASP), exhibits primarily metabolic and endocrine functions, mediated through its C5L2 receptor, with effects on adipocytes, macrophages, and pancreatic cells [38]. C3a is implicated in endometriosis [39,40] and pregnancy loss [40], while elevated C3adesArg levels have been reported in those with polycystic ovary syndrome (PCOS) [41]. In healthy reproductive women, C3adesArg levels are reported to coincide with progesterone levels throughout the menstrual cycle, with low levels across the follicular phase and significantly increased levels in the luteal phase [42,43]. In this study, ovulation was detected using serial transvaginal ultrasonography, which confirmed the presence of the corpus luteum. As progesterone levels were not measured, it is unknown if the UI cohort had progesterone levels that corresponded with C3adesArg levels that would be indicative of effects on the uterine wall lining. Lower levels of C3adesArg would reflect a decrease in C3a or C3 convertase, which may suggest decreased inflammation or immune activation [27]. However, C3a may have an anti-inflammatory role [44] and may modulate adaptive immunity [45]. Whilst C3a was not decreased in this study, C3adesArg was decreased, potentially suggesting a decrease in C3 convertase that may affect the menstrual cycle and endometrial function.

In the classical pathway, C2 associates with activated C4 (C4b) and is cleaved by activated C1 into two fragments, C2a and C2b. C2a combines with factor C4b to generate C3 and C5 convertases. C4 is found in the endometrium, with increased levels associated with inflammation [46] and higher C4 levels in those with primary infertility [22]. Conversely, there is good agreement in the literature that, in women with autoimmune disease, low C4 levels at preconception and during pregnancy are associated with poor obstetric outcomes [47,48]. Similar findings of hypocomplementemia of C4 have been reported in the absence of autoantibodies [49]. Furthermore, the C4 gene was one of the most downregulated genes in the uterus of infertile cows for those who failed to conceive after multiple inseminations despite having normal estrous cycles and no anatomical or infection reproductive abnormalities compared to fertile ones [50]. Thus, a decrease in C4 may result in under activation of this classical pathway or, alternatively, that the decreased levels are due to the immune system actively using C4; notably, a decrease in C4 levels has been associated with infertile patients with endometriosis [51], again suggesting that decreased C4 may reflect endometrial dysfunction.

In the terminal pathway of complement activation, C5 convertase cleaves C5 to C5a and C5b. The C5b bioactive fragment recruits complement components C6, C7, C8, and multiple copies of C9, which together form the potent MAC [37]. The MAC leads to targeted cell death and is protective against infection [52]. C8 is not only essential for the formation of MAC but also plays a protective role by binding the C5b,6,7 complex in the fluid phase, preventing excessive MAC activation [53]. C8 is known to be expressed in the glandular epithelial cells of the endometrium [54], and although the role of C8 in the endometrium is unclear, its deficiency is associated with recurrent infections [55]. In an animal study, C8 subunit gamma has been shown to be present in follicular fluid and to associate with oocyte maturation and fertilization [56]. In this study, the C8 protein was lower in the UI cohort, which may be reflective of changes in the endometrium microenvironment; however, to date, there are no studies in the literature that have investigated levels of C8 in infertile women. While there were no differences between thyroid hormone levels in UI and MFI, in this study, C8 positively correlated with TSH and Free-T3 in UI alone. Research has shown that higher TSH levels in the normal range associate with women with UI compared to those with MFI [57,58] and that lower Free-T3 levels may be a contributing factor to infertility in women with thyroid autoimmunity [59]. Furthermore, thyroid therapy has been shown to alter serum levels of complement proteins, including subunits of C8 [60,61]. Given that thyroid receptors in the endometrium have been shown to play a direct role in endometrial physiology [62], the results here suggest that there may be some interaction between C8 and thyroid hormones that could cause endometrial dysfunction in those with UI.

It is recognized that complement proteins may be altered by hormonal changes including progesterone. However, in this study the confounding effects of hormonal changes through the menstrual cycle were mitigated by all of the women being at day 21 in the luteal phase of their menstrual cycle. In addition, there are no direct studies that associate hormonal levels with the circulating protein levels of C3, C3adesArg, C4, or C8.

Clinically, while biochemical differences were observed, clinical outcomes (fertilization, embryo quality, and live birth rates) were not significantly different in this small cohort. This suggests that these complement alterations may represent early mechanistic differences in endometrial receptivity and follicular development that may not be detected in short-term IVF outcomes but could have implications in natural conception or recurrent implantation failure.

In addition to male and female factors alone, in a third of cases, infertility may be due to a combination of male and female factors [1]. In this study, the MFI group were used as a reference to compare with women classified as having UI, where extensive evaluation, including diagnostic laparoscopy, failed to reveal any underlying cause. Although the possibility of subtle, undetected female factors among the MFI group cannot be completely excluded, the design of the study minimized such overlap. The strengths of the study include the close matching of the MFI and UI cohorts for age and BMI, along with the comprehensive profiling of complement pathway proteins using a state-of-the-art analytical platform. All women were IVF candidates, had discontinued hormonal contraception, abstained from alcohol, and were non-smokers, thereby limiting potential confounding influences. Because complement activity fluctuates with the menstrual cycle [21], this variable was controlled by sampling all participants on day 21 of the cycle.

This study has several limitations including the modest sample size (UI = 14 and MFI = 11), which increases the risk of a type II error given that smaller samples inherently reduce the likelihood of detecting true differences. Limiting the study cohort to a Caucasian population necessitates replication of the study in ethnically diverse populations. In addition, only protein levels were measured in this study. As a result, it was not possible to determine whether differences in protein levels between those with UI and MFI resulted in any changes to functional complement activity. The observational cross-sectional nature also precludes causal inference, and the absence of follow-up data limits the assessment of how complement proteins may vary over time or in response to treatment. It is also possible that other confounders such as subtle endometriosis or subclinical infections that were not detected may have contributed to the findings, and future work in larger, ethnically diverse populations with longitudinal designs, incorporating functional complement activity assays, is required to confirm these preliminary findings and to determine the biological mechanisms underlying the observed differences. Such studies would provide more robust insights into potential subtle differences and enhance the generalizability of these findings.

In conclusion, the findings reported here indicate reduced complement activation signatures in women with unexplained infertility, suggesting potential pathways for future biomarker validation and therapeutic exploration.

## 4. Materials and Methods

### 4.1. Study Design and Participants

This case–control study was conducted in 2015 at the IVF Unit, Hull, UK. Twenty-five women were recruited sequentially. Ethical approval was granted by the Yorkshire and The Humber NRES Ethics Committee, UK (approval number 02/03/043) [63]. Exclusion criteria included the following: documented immunological or inflammatory disease, acute or chronic infection, hepatic or renal insufficiency, diabetes mellitus, BMI > 30 kg/m^2^, age outside of the 20–45-year range, and individuals not undergoing IVF treatment. Medical history review confirmed that none of the participants were taking prescription or over-the-counter medications, all were non-smokers, and all had abstained from alcohol for more than six months. Women with unexplained infertility (UI) underwent diagnostic laparoscopy as part of their evaluation.

### 4.2. IVF Protocol

All participants commenced IVF treatment during the subsequent menstrual cycle using a short antagonist protocol. Recombinant follicle-stimulating hormone (rFSH) stimulation was initiated on day 2 of the cycle with either Merional (Pharmasure, Watford, UK) or Gonal-F (Merck Serono, Feltham,, UK). The dose was individualized according to AMH levels, antral follicle count, age, and ovarian response to prior treatment. From day 6 of stimulation, premature luteinizing hormone (LH) surge was prevented using Cetrotide (GnRH antagonist; Merck Serono, Feltham,, UK) at 0.25 mg/day. Final oocyte maturation was triggered when ≥2 leading follicles reached ≥18 mm, using either 0.5 mg Buserelin (Sanofi-Aventis, Frankfurt, Germany) or 5000–10,000 IU human chorionic gonadotrophin (hCG; Pregnyl, Merck Sharp and Dohme, London, UK). Oocyte retrieval was performed transvaginally 36 h later. Luteal phase support was initiated on the day of oocyte retrieval with vaginal progesterone (Uterogestan, Besins Iscovesco Laboratories, Paris, France; 600 mg nightly). Embryo transfer was carried out on day 3 or preferably day 5 (blastocyst stage) to maximize implantation potential. Embryos were graded at both cleavage and blastocyst stages using standardized morphological criteria [64].

### 4.3. Sample Collection

Ovulation was detected using serial transvaginal ultrasonography, which confirmed the presence of the corpus luteum, and at day 21 of the menstrual cycle, before initiation of IVF treatment and before administration of any hormonal therapy, participants underwent mock embryo transfer (standard clinical practice) and ovarian/endometrial ultrasound. Fasting blood samples were collected, centrifuged (3500× *g* for 15 min at 4 °C), and stored at −80 °C until analysis.

### 4.4. Biochemical and Hormonal Assays

#### 4.4.1. Metabolic Measures

Fasting blood glucose (FBG) was measured using a Synchron LX20 analyzer (Beckman-Coulter, High Wycombe, UK). Total cholesterol, triglycerides, and HDL-c were measured enzymatically using a Synchron LX20 analyzer (Beckman-Coulter). Low-density lipoprotein cholesterol (LDL-c) was calculated using the Friedewald equation [65]. Serum insulin concentrations were determined by competitive chemiluminescent immunoassay (Immulite 2000, Euro/DPC, Llanberis, UK). Insulin resistance was assessed using the HOMA-IR, calculated as (insulin × glucose)/22.5 [66]. Glycosylated hemoglobin (HbA1c) was determined by ion-exchange chromatography. CRP was evaluated using enzymatic assays on the Synchron LX20 analyzer (Beckman-Coulter). WCC was measured with a Beckman Coulter counter (Beckman Coulter).

#### 4.4.2. Reproductive Hormones

AMH levels were determined with an immunoenzymatic assay (Beckman-Coulter). Circulating androgens were quantified by liquid chromatography–tandem mass spectrometry (LC/MS/MS; Acquity UPLC-Quattro Premier XE-MS, Waters, Manchester, UK). Sex hormone-binding globulin (SHBG) was assessed using an immunometric fluorescence assay (Immulite 2000 analyzer; upper limit 2.0 nmol/L). The Free Androgen Index (FAI) was calculated according to the formula (testosterone/SHBG) × 100. Thyroid hormone levels, TSH, Free-T3, and Free-Thyroxine (Free-T4) were determined using an immunoassay on the Abbott Architect i4000 platform (Abbott Diagnostics, Maidenhead, UK).

#### 4.4.3. Complement Protein Quantification

Complement plasma proteins were quantified using the Slow Off-rate Modified Aptamer (SOMAscan) platform, version 3.1 (SomaLogic, Boulder, USA), as previously described [67,68]. In brief, synthetic SOMAmers with fluorescent labeling were first bound to analyte/primer beads, and the resulting complexes were immobilized on a streptavidin matrix. Ultraviolet (UV) light was applied to cleave the photocleavable linker, releasing the analyte–SOMAmer complexes into solution. These complexes were subsequently re-immobilized on a streptavidin matrix through analyte-mediated biotinylation, after which the SOMAmers were eluted and used as surrogates for protein quantification. Quantification was achieved through hybridization with complementary oligonucleotides, enabling accurate signal detection. Calibration standards were included, and normalization procedures—comprising hybridization control, median signal scaling, and calibration signal correction—were applied in accordance with established protocols: intra-plate CV: plasma ~3.6%; inter-plate CV: plasma ~3.8%; and total CV: plasma ~5.3%. [69,70]. The complement and related proteins analyzed were properdin, C3b, inactivated C3b (iC3b), C3, C3 anaphylatoxin (C3a), C3adesArg, C3d, C4, C4a, Factor I, Factor D, C2, complement factor H-related protein 5 (CFHR5), Factor B, Factor H, C5a, complement C5b-C6 complex (C5b, 6 Complex), C5, C1q, C1r, mannose-binding lectin (MBL), mannan-binding lectin serine protease 1 (MASP3), complement decay accelerating factor (DAF), C4b, and C8.

### 4.5. Statistical Analysis

As this was an exploratory pilot, no formal power calculation was performed, but *n* = 25 was selected to estimate effect sizes for future studies. The mean ± standard deviation (SD) was presented as descriptive data for continuous data. For the values of complement proteins and metabolic or hormonal levels, normality was assessed using the Kolmogorov–Smirnov (K-S) statistical test; for all measurements, the K-S *p*-value was >0.05, indicating that the data were normally distributed. Student’s *t*-test was used to compare the differences between groups, and the result was determined to be statistically significant if *p* < 0.05. The false discovery rate was employed to account for multiple tests. Associations between endothelial protein levels and metabolic and hormonal parameters were calculated by Pearson’s correlations. GraphPad Prism v10.4.1 (San Diego, CA, USA) was used for statistical analysis and visualization.

## Figures and Tables

**Table 1:** Demographic and biochemical data compared between male factor infertility (n=14) and unexplained infertility (n=11). The data is presented as mean (SD). The p values were calculated using Student’s t-test to determine any differences between the groups.

	MFI	UI	*p* Value
Age (years)	32.6 (4.0)	33.8 (5.3)	0.51
BMI (kg/m^2^)	25.7 (2.6)	25.3 (4.9)	0.84
HOMA-IR	1.5 (0.7)	1.9 (1.4)	0.45
Cholesterol (mmol/L)	4.8 (0.8)	4.6 (0.7)	0.44
Triglycerides (mmol/L)	0.9 (0.5)	1.0 (0.4)	0.67
HDL-c (mmol/L)	1.6 (0.4)	1.6 (0.2)	0.97
LDL-c (mmol/L)	2.8 (0.7)	1.0 (0.4)	0.24
CRP (mg/L)	1.9 (1.3)	2.4 (2.0)	0.51
WCC × 10^9^/L	5.9 (1.7)	7.2 (2.2)	0.12
AMH (ng/mL)	22.4 (15.3)	24.5 (12.5)	0.72
FAI	1.4 (0.7)	0.8 (0.9)	0.11
TSH (mU/L)	2.3 (1.2)	2.0 (0.8)	0.53
Free-T3 (pmol/L)	4.7 (0.7)	4.8 (0.6)	0.66
Free-T4 (pmol/L)	11.2 (1.6)	11.4 (0.9)	0.66
Positive pregnancy test	0.3 (0.5)	0.3 (0.5)	0.95
Number of eggs retrieved	9.0 (7.5)	8.4 (3.2)	0.78
Number of embryos created	3.7 (3.0)	5.2 (2.4)	0.18
G3D3	3.4 (2.2)	2.7 (2.6)	0.49
Fertility rate	0.6 (0.2)	0.6 (0.4)	0.89
Top quality embryo (proportion)	0.3 (0.2)	0.4 (0.4)	0.36
Live birth rate	0.0 (0.0)	0.0 (0.0)	1.00

BMI, body mass index; HOMA-IR, Homeostatic Model Assessment of Insulin Resistance; HDL-c, High-Density Lipoprotein Cholesterol; LDL-c, Low-Density Lipoprotein Cholesterol; CRP, C-Reactive Protein; WCC, white cell count; AMH, Anti-Müllerian Hormone; FAI, Free Androgen Index; TSH, thyroid-stimulating hormone; Free-T3, Free-triiodothyronine; Free-T4, Free-Thyroxine; and G3D3, Top-Quality Day 3 Embryos.

**Table 2:** Comparison of plasma levels of complement pathway proteins in male factor infertility (MFI) and unexplained infertility (UI). The data is presented as mean (SD). Protein levels are reported as Relative Fluorescence Units (RFUs).

	MFI	UI	*FDR p* Value
Properdin	131,606 (23,723)	91,387 (61,357)	0.034 #
C3b	43,399 (25,396)	45,084 (54,190)	0.92
iC3b	6073 (1129)	5580 (1806)	0.41
C3	48,348 (6443)	40,796 (25,267)	0.29
C3adesArg	137,187 (13,467)	99,554 (56,569)	0.024 #
C3a	399 (49)	402 (142)	0.94
C3d	8789 (2301)	6551 (3966)	0.09
C4	117,538 (25,704)	95,901 (21,341)	0.04 #
C4a	72,800 (2391)	73,259 (2544)	0.65
Factor I	41,056 (4648)	38,334 (3178)	0.11
Factor D	722 (83)	639 (208)	0.19
C2	2874 (194)	2870 (289)	0.96
CFHR5	1613 (756)	1331 (442)	0.25
Factor B	28,997 (4571)	28,385 (3906)	0.73
Factor H	60,035 (5505)	57,943 (5549)	0.36
C5a	11,127 (3728)	10,942 (5900)	0.93
C5b, 6 Complex	491 (31)	501 (61)	0.61
C5	6990 (461)	7117 (1130)	0.71
C1q	35,166 (6834)	33,787 (6928)	0.62
C1r	2783 (982)	3225 (983)	0.28
MBL	15,048 (8231)	11,240 (5960)	0.21
MASP3	5791 (782)	5275 (1348)	0.24
DAF	14,938 (2131)	13,127 (3505)	0.12
C4b	261 (122)	417 (255)	0.06
C8	1974 (307)	1609 (528)	0.04 #

C1–C8, complement component 1–8; iC3b, inactivated C3b; C3adesArg, C3a anaphylatoxin des arginine; C3a, C3 anaphylatoxin; C4a, C4 anaphylatoxin; CFHR5, complement factor H-related protein 5; C5a, C5 anaphylatoxin; C5b, 6 Complex, complement C5b-C6 complex; MBL, mannose-binding lectin; MASP, mannose-associated serine protease; and DAF, decay-accelerating factor. #, *p* < 0.05.

## Data Availability

The data that support the findings of this study are available from the corresponding author upon reasonable request.

## Abbreviations

The following abbreviations are used in this manuscript:

IVF	In Vitro Fertilization
UI	Unexplained Infertility
MFI	Male Factor Infertility
SOMA	Slow Off-rate Modified Aptamer
C3adesArg	C3a Anaphylatoxin
MBL	Mannose-Binding Lectin
MAC	Membrane Attack Complex
rFSH	Recombinant Follicle-Stimulating Hormone
AMH	Anti-Müllerian Hormone
ASP	Acylation-Stimulating Protein
PCOS	Polycystic Ovary Syndrome
BMI	Body Mass Index
HOMA-IR	Homeostatic Model Assessment of Insulin Resistance
HbA1c	Glycosylated Haemoglobin
CRP	C-Reactive Protein
WCC	White Cell Count
LH	Luteinizing Hormone
FBG	Fasting Blood Glucose
HDL-c	High-Density Lipoprotein Cholesterol
LDL-c	Low-Density Lipoprotein Cholesterol
SHBG	Sex Hormone-Binding Globulin
FAI	Free Androgen Index
TSH	Thyroid Stimulating Hormone
Free-T3	Free-Triiodothyronine
Free-T4	Free-Thyroxine
iC3B	Inactivated C3b
C3a	C3 Anaphylatoxin
C3adesArg	C3a Anaphylatoxin Des Arginine
CFHR5	Complement Factor H-Related Protein 5
C5a	C5 Anaphylatoxin
C5b, 6 Complex	Complement C5b-C6 Complex
MASP3	Mannan-Binding Lectin Serine Protease 1
DAF	Complement Decay-Accelerating Factor
